# Editorial: Women in retina: 2022

**DOI:** 10.3389/fopht.2023.1169163

**Published:** 2023-03-15

**Authors:** Genea Edwards, Stephanie L. Grillo

**Affiliations:** ^1^ Department of Ophthalmology, Vision Research Center, School of Medicine, University of Missouri-Kansas City, Kansas City, MO, United States; ^2^ Department of Ophthalmology, Pennsylvania State University-Milton S. Hershey Medical Center, Hershey, PA, United States

**Keywords:** diabetic complications, age-related macular degeneration, parallel processing, choroideremia, ocular hypertension, biomarkers

The goal of the inaugural Frontiers in Ophthalmology, *Women in Retina: 2022* collection of articles was to offer a platform in which to promote women scientists from all specialties of retinal research. Each year on February 11^th^ we observe International Day of Women and Girls in Science, to raise awareness and boost funding programs to promote women in science. The success of women in Science, Technology, Engineering, and Mathematics (STEM) fields serves as a much-needed inspiration to all scientists and to support one another in our lives and careers.

The retina’s neural circuitry is a highly organized extension of the brain and central nervous system, with an underlying uveal layer of vascular and connective tissue. It is essential to understand the pathophysiology of diseases that cause neurodegeneration of the retina, such as diabetic retinopathy, age-related macular degeneration, choroideremia, and ocular hypertension. This will aid future research to spearhead advances in diagnostics, treatment, and therapeutic targets for protecting one of our most important senses, vision.

Contribution toward a broad range of complementary research efforts led by women, culminated in three reviews and three original research articles:

Prolonged hyperglycemia has been shown to cause long-term visual complications and an increased risk of cognitive defects. It was reported by Rowe et al. that there is time- and tissue-specific effects of prolonged glucose exposure in hyperglycemic zebrafish. Their results suggest that inflammation is an early cellular response to increased blood sugar levels. Glucose-specific differences in tight junction and neuronal markers detected at 4 weeks became osmotically driven at 8 weeks. This suggests there is either the development of a secondary osmotic effect caused by hyperglycemic exposure or that zebrafish may be able to adapt and compensate for the prolonged glucose insult.

A study utilizing Optical Coherence Tomography Angiography (OCTA) to detect choriocapillaris’ vessel density (CVD) at different stages of age-related macular degeneration (AMD) by Savastano et al. reported that OCTA could play a crucial role in the categorization of AMD, allowing for the assessment of gradually diminished blood flow at different stages of the disease. Their results highlight statistically significant differences between early AMD and both late AMD and disciform scar AMD subgroups. These findings could potentially to the development of an important diagnostic biomarker for AMD pathology.

A comprehensive review by Ichinose and Habib nicely summarized the role of ON-OFF neural signaling pathways in visual processing. Emerging evidence has indicated that the visual system is comprised of an elaborate array of neural networks that analyze visual signals in the retina, which are subsequently sent to the brain for visual processing. Recognition of visual signals is accomplished by parallel processing, where multiple features of visual signals are encoded into separate neural streams and analyzed in parallel. ON and OFF pathways (occurring from bipolar cell responses) had been thought to signal in a mirror-symmetric fashion. In some cases they have distinct asymmetries, suggesting an independent operation while both contribute synergistically to visual perception.

Choroideremia (CHM) is a rare X-linked recessive retinal degenerative disease characterized by a gradual loss of night vision. A study by Edwards et al. investigated glial remodeling and activation along with choriocapillaris changes and their association with retinal pigment epithelium (RPE) loss by histopathology. A dense glial membrane comprised of vimentin and GFAP was observed occupying the subretinal space in the area of RPE and photoreceptor cell loss. A glial membrane was also found on the vitreoretinal surface. Furthermore, analysis detected glial cell disorganization, containing exosome-like vesicles and a total absence of choriocapillaris in areas of RPE loss. The far periphery of the donor eyes showed no glial membrane with intact RPE and viable choroidal vessels. These findings prove vital for identifying new treatments for choroideremia.

A short review exploring the impact of acute intraocular pressure (IOP) on retinal ganglion cell (RGC) injury and recovery by Garner et al. emphasized the need for therapeutic interventions in glaucoma to promote RGC survival. In this review, the authors highlighted the morphological and molecular responses of axons and dendrites of RGCs and retinal glia by acute, multi-factorial IOP insult as compared to more chronic models of elevated IOP. Interruption to axonal transport, up-regulation of markers for cell proliferation, and down-regulated pathways involved in axon extension, dendrite morphogenesis, and metabolism of RGCs preceding the onset of cell death by apoptosis are shown to occur. Glial inflammatory responses may produce a protective response initially, by releasing antioxidants and neurotrophic factors, but RGC excitotoxicity can occur from prolonged reactivity of astrocytes and microglia.

While there is no established treatment for dry AMD and limited success from therapeutics for wet AMD, the short review by Cruz-Aguilar et al. delved into the use of miRNAs as potential biomarkers or therapeutic targets. Dysregulation in miRNA expression of AMD in animals and humans can be found in processes involving oxidative stress, inflammation, and angiogenesis. Identification of miRNAs specific to different types and stages of AMD is currently under investigation in clinical studies. Molecular studies using plasma, serum, vitreous humour, blood, and retinal tissue have all detected aberrant miRNAs in samples from AMD patients and warrant further investigation for understanding their importance.

In conclusion, the Frontiers Women in Retina: 2022 Research Topic showcased women’s contribution to current advances in retinal research. The wide variety of topics presented above is sure to encourage and promote the future growth of women and girls in STEM careers.

## Author contributions

Editorial was written by GE and SG. Both authors contributed, edited, reviewed, and approved the submitted version for publication.

